# Deubiquitinating Enzyme: A Potential Secondary Checkpoint of Cancer Immunity

**DOI:** 10.3389/fonc.2020.01289

**Published:** 2020-08-07

**Authors:** Xing Huang, Xiaozhen Zhang, Jian Xu, Xun Wang, Gang Zhang, Tianyu Tang, Xiaochao Shen, Tingbo Liang, Xueli Bai

**Affiliations:** ^1^Zhejiang Provincial Key Laboratory of Pancreatic Disease, The First Affiliated Hospital, School of Medicine, Zhejiang University, Hangzhou, China; ^2^Department of Hepatobiliary and Pancreatic Surgery, The First Affiliated Hospital, School of Medicine, Zhejiang University, Hangzhou, China; ^3^Innovation Center for the Study of Pancreatic Diseases, Hangzhou, China

**Keywords:** cancer immunotherapy, deubiquitination, deubiquitinating enzymes, immune checkpoint, secondary checkpoint

## Abstract

The efficacy of cancer immunotherapy depends on the fine interplay between tumoral immune checkpoints and host immune system. However, the up-to-date clinical performance of checkpoint blockers in cancer therapy revealed that higher-level regulation should be further investigated for better therapeutic outcomes. It is becoming increasingly evident that the expression of immune checkpoints is largely associated to the immunotherapeutic response and consequent prognosis. Deubiquitinating enzymes (DUBs) with their role of cleaving ubiquitin from proteins and other molecules, thus reversing ubiquitination-mediated protein degradation, modulate multiple cellular processes, including, but not limited to, transcriptional regulation, cell cycle progression, tissue development, and antiviral response. Accumulating evidence indicates that DUBs also have the critical influence on anticancer immunity, simply by stabilizing pivotal checkpoints or key regulators of T-cell functions. Therefore, this review summarizes the current knowledge about DUBs, highlights the secondary checkpoint-like role of DUBs in cancer immunity, in particular their direct effects on the stability control of pivotal checkpoints and key regulators of T-cell functions, and suggests the therapeutic potential of DUBs-based strategy in targeted immunotherapy for cancer.

## Background

**Figure 1:**
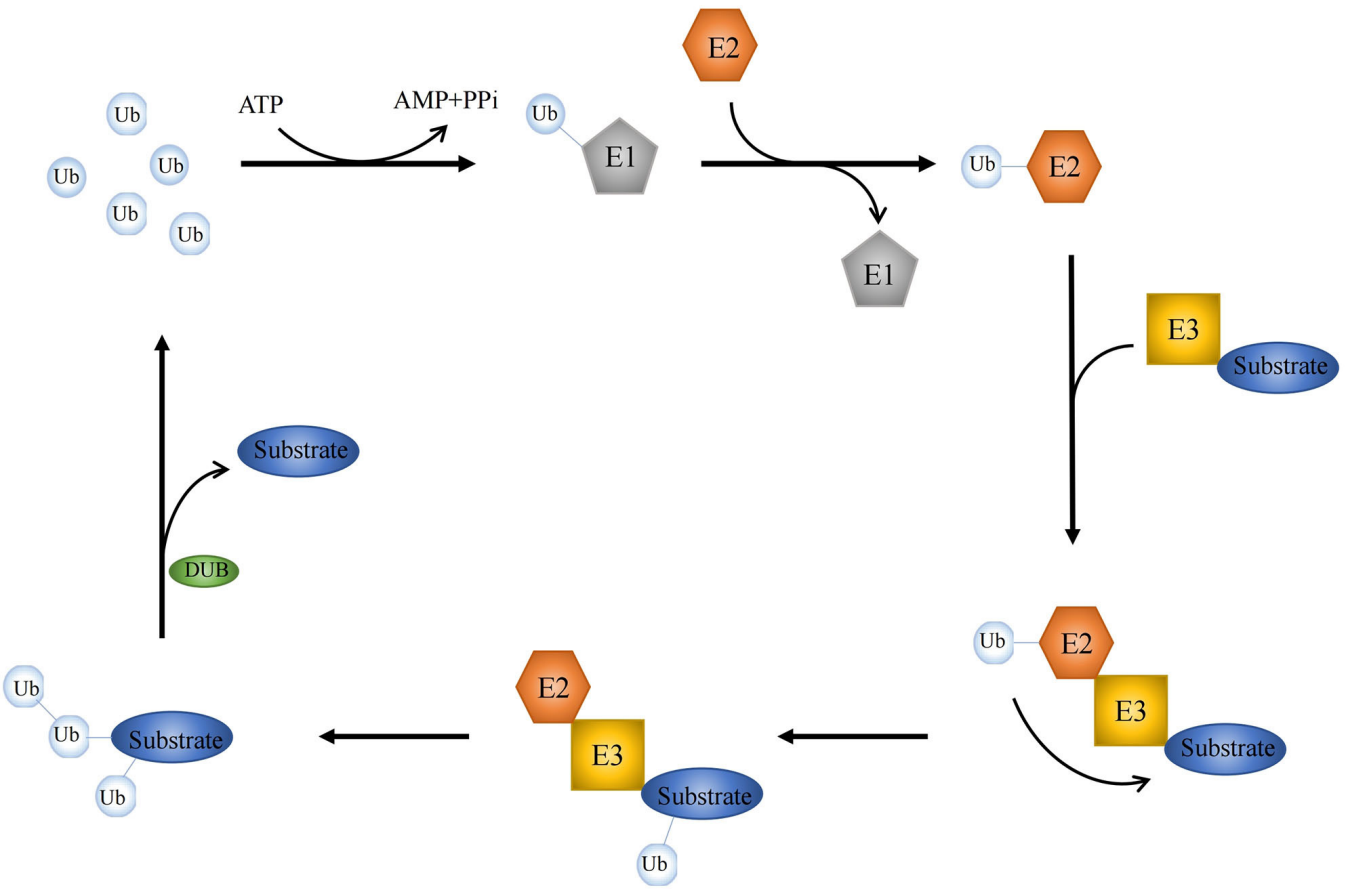
Key events in ubiquitylation and deubiquitylation. A schematic illustration of three-step enzymatic process in ubiquitination. E1, E2, and E3 form a covalent bond to coordinate the editing between ubiquitin and its substrate protein, while DUBs reverse this processing.

As one kind of posttranslational modification, ubiquitination is mediated by a series of enzymatic reactions, mostly initiating protein degradation and thus affecting protein stability (1, 2). Besides, more and more ubiquitination-dependent but protein degradation-irrelevant events are discovered, and further studies indicate that a number of vital cellular processes are triggered by ubiquitination (3, 4). Thus, the ubiquitin system is complex and vast, affecting every aspect of cell life. There are a great many types of ubiquitination, but the dominant forms are monoubiquitination, and Lys48 or Lys63-linked polyubiquitination (5). The ubiquitin signal is modulated by an enzymatic cascade involving two ubiquitin-activating enzymes (E1), ~40 ubiquitin-binding enzymes (E2), and more than 700 ubiquitin ligases (E3), as well as ~100 of deubiquitinating enzymes (DUBs) (6–8). Ubiquitination is a highly conserved and tightly controlled enzymatic process with three joint steps. E1, E2, and E3 work together to form a covalent bond between ubiquitin and its substrate protein for chain editing and precursor processing to complete the processing of ubiquitin, whereas DUBs reverse this progression (Figure 1).

Deubiquitinating enzymes are proteolytic enzymes that can cleave ubiquitin or ubiquitin-like proteins from their target substrate or proproteins, thus inhibiting ubiquitination-mediated protein degradation (9, 10). Based on sequence and domain conservation, six DUB families with distinct structures have been described. Families of cysteine peptidases include ubiquitin-specific proteases (USPs), ubiquitin COOH terminal hydrolases (UCHs), Machado-Josephine domain-containing proteases, ovarian tumor–associated proteases (OTUs), zinc finger–containing ubiquitin peptidases, and motif interacting with ubiquitin-containing novel DUB family (MINDY). In addition, a family of Zn-dependent peptidase JAB1/MPN/MOV34 metalloprotease DUBs (JAMMs, also known as MPN+; 16 members) exists (11, 12). Other protease classes, such as ubiquitin-like proteases, also act as protein-like modifiers, including, but not limited to, SUMO [Sentrin/SUMO-specific protease (SENP) and de-SUMO-glycosylated isopeptidase (DeSI) family] and NEDD [NEDD8-specific protease 1 (NEDP1), member of the SENP family] (13, 14). For instance, the deISGylating enzyme USP18 is a ubiquitin-like protease that plays a key role in the innate immune system. USP18 acts as an endogenous isopeptidase that cleaves the ubiquitin-like ISG15, which is the representative type I interferon-induced gene and also the first identified ubiquitin-like protease (15, 16).

As mentioned above, multiple modes regulate DUB interaction with ubiquitin and substrate, enabling multiple mechanisms to fine-tune DUB function. The mechanism used by cells to regulate DUB function can be roughly divided into two main types: one is to regulate DUB abundance and position, and the other is to regulate its catalytic activity (17, 18). The catalytic domain of DUBs can directly bind to its substrates and further recognize the specific ubiquitin sites, which determines the activity and specificity of DUBs. All types of DUB classes have at least one ubiquitin binding site, called S1 site, which guides the ubiquitin C-terminus and the frangible bond to the active site, followed by hydrolysis. When double ubiquitin, the distal ubiquitin occupies the S1 site, while the proximal ubiquitin occupies the S1′ site. Moreover, some of DUBs have extra ubiquitin binding sites such as S2, S3, S2′, or S3′, which allow the polymerized ubiquitin chain binding to precise position in enzymes and thus may contribute to the specificity of connection (11, 17).

In addition to conventional effects on the protein stability and expression level, accumulating evidence suggested that the ubiquitin-based regulatory system also plays a crucial role in immunological process. This review hereafter summarizes the novel roles of DUBs and deubiquitination on protein-dependent antitumor immune responses, majorly focusing on different immune cell signaling cascades, including, but not limited to, the tumor necrosis factor (TNF) signaling cascade in T cells and B cells.

## Effect of DUBs on Tumoral Immune Checkpoints

**Figure 2:**
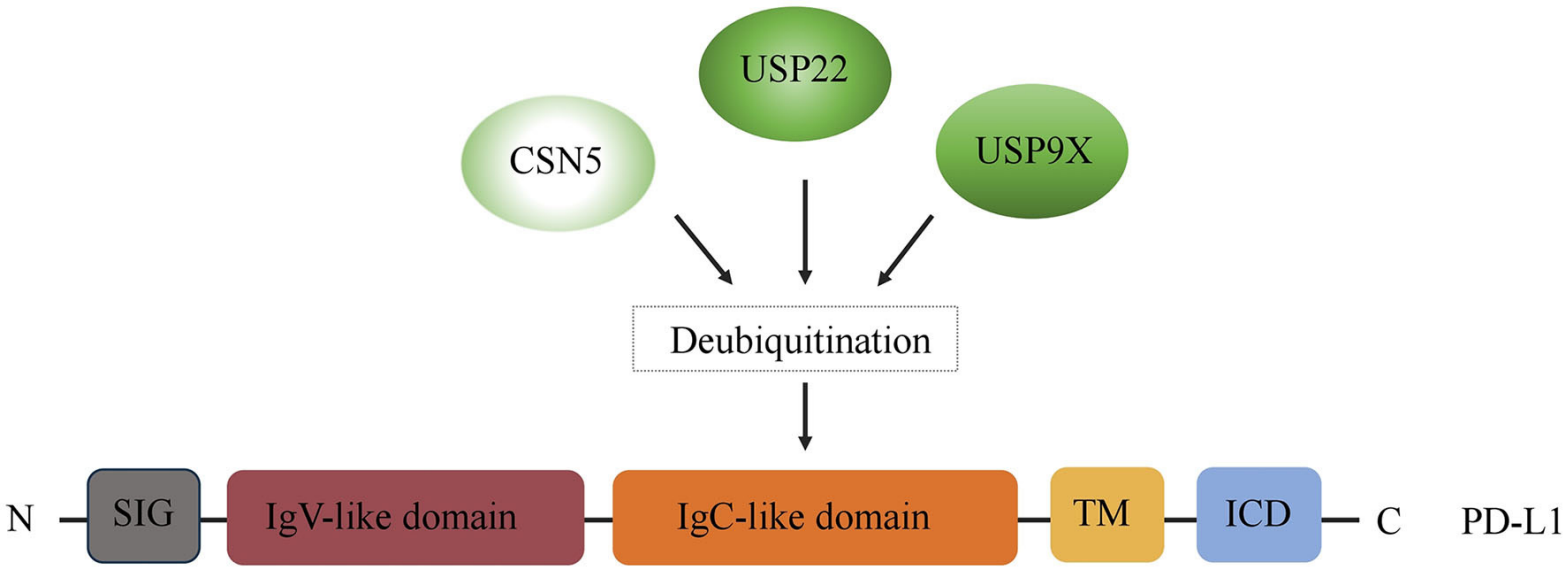
Regulation of PD-L1 by DUBs. A schematic illustration of multiple DUBs involved in PD-L1 deubiquitylation, including USP22, CSN5, and USP9X. ICD, intracellular domain; TM, transmembrane domain; EC, extracellular domain; SIG, signal sequence.

Immune checkpoints are part of the immune system. Their role is to prevent a strong immune response and the destruction of healthy human cells by the immune system itself. Immune checkpoints [including Programmed Cell Death Protein 1 (PD-1) and Cytotoxic T-Lymphocyte-Associated Protein 4 (CTLA-4)] work when proteins on the surface of immune cells such as T cells recognize and bind to chaperone proteins (such as PD-L1) on other cells, including certain tumor cells. Immune checkpoint blockade–based immunotherapy provides novel and promising approaches for cancer patients, which may result in a long-time control of the tumor or even cure it (19, 20). However, the immune checkpoint inhibitors (such as PD-1/PD-L1 antibody drugs) did not achieve the expected efficacy in the treatment of certain tumors (e.g., pancreatic cancer) (21). It is well-known that the expression of the target is needed for the corresponding therapy to work. For instance, PD-L1 expression, on tumor cells and/or T cells, is a prognostic factor for PD-1/PD-L1–targeting immunotherapies. Accumulating evidence suggests that the expression of immune checkpoints is regulated at multiple levels from different levels. Recently, increasing evidence indicates that immune checkpoints are also regulated by multiple posttranslational modifications in tumors, thus regulating their ability to mediate immune escape (22). In this context, DUB has become a crucial factor in ubiquitination and deubiquitination, which is a type of posttranslational modification. Thus, in the first part of our review, we summarize the recent findings regarding the regulatory effects of three key DUBs, USP22, CSN5, and USP9X, on PD-L1, the most representative immune checkpoint (Figure 2).

### USP22

USP22 is a novel human DUB composed of 525 amino acids, and containing Cys, Asp, His, and Asp/Asn, which are highly conserved domains of the UBP family in DUBs (23). Some results show that USP22 is overexpressed in many tumor types and affects tumorigenesis and development by affecting cell cycle (24–28). However, our previous study demonstrated that USP22 induces the deubiquitination of PD-L1 and prevents PD-L1 degradation. Binding directly to the C-terminal cytoplasmic tail and transmembrane region of PD-L1, USP22 catalyzes the deubiquitination of PD-L1 and stabilizes PD-L1 in a CDK4-independent manner (29). Hepatocellular carcinoma is the tumor that better fits in the exploration of tumor inhibition mediated by USP22–PD-L1, because the expression of USP22 in this tumor is higher than its expression in other cancer types (30). In pancreatic cancer, in addition to affecting tumor progression through the regulation of the cell cycle, USP22 can also influence tumorigenesis and development by regulating immune cell infiltration through nuclear functions independent of its effects on PD-L1 protein stability (31). Although the expression of PD-L1 is a predictive biomarker for the result of immunotherapy in patients with multiple tumor types, the therapeutic effect of targeting PD-L1 is unsatisfactory (21, 32, 33). Our hypothesis is that the poor clinical efficacy of the treatments targeting PD-L1 may be due to the stabilization of PD-L1 mediated by USP22, abolishing the effect of anti–PD-L1 drugs (30). Therefore, a targeted inhibition of USP22-mediated deubiquitination of PD-L1 can be a new potentially effective immunotherapeutic strategy.

### CSN5

COP9 signalosome subunit 5 (CSN5), which is also called JAB1, is the fifth component of the CSN regulatory complex and contains an evolutionarily conservative Jab1/Mpr1p and Pad1pN terminus (MPN) domain metalloenzyme (JAMM) motif interface with the ubiquitin–proteasome pathway. CSN5 plays critical roles in regulating the invasion and migration of cancer cells, as well as exosome protein sorting (34–37). Some results by mass spectrometry analysis revealed that CSN complex is the interaction partner of PD-L1 (34, 38, 39). Acting as a DUB, CSN5 inhibits the ubiquitination processing and subsequent proteasomal degradation of PD-L1 and thus stabilizing its protein expression in cancer cells. Tumor necrosis factor α stimulation activates nuclear factor κB (NF-κB), which induces CSN5 expression, resulting in PD-L1 stabilization. However, the CSN5 inhibitor curcumin inhibits the stability of PD-L1, making cancer cells sensible to an anti-CTLA4 therapy (38). In colorectal cancer, Liu et al. (39) suggested that the C-C motif chemokine ligand 5 (CCL5), secreted from macrophages, inhibits T cell–mediated killing in HT29 cells. Mechanistically, CCL5 promotes the immune escape through causing the formation of STAT3/NF-κB p65 complex, which bound to the *CSN5* promoter, and in turn modulates the deubiquitination and stability of PD-L1 *in vitro* and *in vivo* (39).

### USP9X

Previous studies showed that the function of USP9X in regulating cancer cells is complex and diverse. Through a ubiquitin-specific protease activity, USP9X not only plays a paramount role in regulating the proliferation, apoptosis, and adhesion of cancer cells (40, 41), but also maintains the stability of DNA replication-fork and DNA-damage checkpoint responses, thus affecting radiosensitivity (42, 43). Recently, it has been revealed that USP9X also plays a vital role in regulating immune checkpoints. Indeed, USP9X induces PD-L1 deubiquitination and regulates its stabilization by ubiquitin specific protease activity (44). In Oral Squamous Cell Carcinoma (OSCC) cells, the high expression of USP9X increases the deubiquitination of PD-L1 and reduces its degradation, resulting in protein accumulation in these cells (44). Thus, targeting PD-L1 by blocking USP9X may be a potentially useful strategy in the treatment of cancer cells.

## Role of DUBs in T Cell Function and Immune Response

Ubiquitination is one kind of critical mechanism in regulating the immune response and T-cell function. Although the full-scale roles of DUB in immunity have not been thoroughly understood, significant progresses have recently been reported by some studies regarding immunoregulation by DUBs. In the following part, we discuss the up-to-date findings on the molecular features and signaling function of deubiquitylation in immune response and T-cell function (Table 1). There are many DUBs regulating the immune response, majorly from USP family, including CYLD, USP4, USP7, USP8, USP9X, USP11 USP15, USP17, USP18, USP25, and USP47, as well as OTU family, including A20, deubiquitylating enzyme A (DUBA), Otud7b, OTULIN, BRCC/ABRO, and Zranb1. Specifically, USP11, A20, and OUTLIN contribute to immune response through NF-κB pathway, whereas USP25 regulates innate immunity by deubiquitylation of the adaptor protein TRAF3 (45–49). Moreover, USP7, USP47, and BRCC3/ABRO can activate the NLRP3 inflammasome (50–54). Furthermore, USP4, USP15, USP17, and USP18 control T_H_17 differentiation through RORγt pathway. Additionally, USP18 is a key regulator of interferon signaling and its mediated innate immune response (45, 55–58). Because the regulatory mechanism of DUBs in immunity is quite complex, we focus on the representative ones in this part.

**Table 1:** Deubiquitinases involved in immune response and T-cell regulation.

**Family**	**DUB**	**Function**	**Target**
USP	CYLD	Survival of immature NKT cells	IKK
		T cell activation	TAK1/IKK
		Thymocyte development	LCK
		Treg development	IKK/Smad7
	USP4	T_H_17 differentiation	RORγt
	USP7	Treg function	Foxp3/ Tip60
		inflammasome activation	NLRP3
	USP8	Thymocyte maturation	CHMP5
	USP9X	TCR signaling and central tolerance	Bcl10/Zap70/Themis
	USP10	Unknown	T-bet
	USP11	Immune response	NF-κB pathway
	USP15	T-cell activation and differentiation	MDM2
		T_H_17 differentiation	RORγt
	USP17	T_H_17 differentiation	RORγt
	USP18	T_H_17 differentiation	RORγt
		Innate immune response	IFN-γ pathway
	USP25	Innate immune response	TLR pathway
	USP47	Inflammasome activation	NLRP3
OTU	A20	NKT cell differentiation	MALT1
		CD4 T-cell survival	RIPK3
		T-cell survival	mRORC1
		CD8 T-cell activation/Treg development/cell-extrinsic regulation of T_H_1 and T_H_17 cell differentiation	NF-κB pathway
	DUBA	T-cell activation and differentiation	UBR5
	Otud7b	T_H_17 differentiation	Zap70
	OTULIN	Innate immune response	NF-κB pathway
	BRCC3/ABRO	Inflammasome activation	NLRP3
	Zranb1	Cell-extrinsic regulation of T_H_1 and T_H_17 cell differentiation	Jmjd2b

### A20

A20, also known as TNFAIP3, is a zinc finger domain–containing deubiquitinase that limits the function of TNF receptor (TNFR) and induces NF-κB activation via innate immune receptors, such as Toll-like receptors (TLRs) and NOD2 (nucleotide-binding oligomerization domain protein 2, an intracellular pattern recognition molecule) (59–62). A unique feature of A20 is not only its capability to act as a DUB, but also works as an E3 ligase (59). The amino-terminal (N-terminal) region of A20 contains an OUT (the ovarian tumor–related proteases) domain, which is composed of ~24 members in the human genome and forms the second largest DUB family in mammalian. A20 has DUB activity toward several NF-κB signaling factors that regulate both innate and adaptive immune responses. In addition, A20 inhibits receptor-interacting protein kinase 1 (RIP1, also called RIPK1) K63-linked polyubiquitination to block NF-κB signaling downstream of TNFR1 (59, 62, 63). Of note, the carboxy-terminal domain of A20 contains seven C2/C2 zinc finger domains; thus, it is able to act as an E3 ubiquitin ligase. For instance, A20 can polyubiquitinate RIP with K48-linked ubiquitin chains, thereby promoting the proteasomal degradation of RIP (59).

In addition to the TNFR pathway, the function of A20 has been described in the IL-1R/TLR4 pathway. A20 targets E3 ligases via being recruited to the E3 ligases TRAF2, TRAF6, and cIAP1/2, disrupting the connection between E2 and E3 ubiquitin enzyme complex, and destroying the interactions with the E2 enzymes Ubc13 and UbcH5c. The ubiquitination and degradation of the E2 enzymes occur at later time points after stimulation (64). A20 was the first DUB found to have a role in innate immune regulation. Some studies have shown that the loss of A20 leads to continued activation of NF-κB by TLRs and TNFR by breaking the tolerance of the innate immune system to the commensal intestinal microflora, causing abnormal homeostatic TLR signaling and the production of pro-inflammatory mediators (60, 62, 65). Therefore, A20 is a key protein that regulates immune homeostasis and TLR signaling *in vivo*. In different immune tissues, the important role of A20 has been described using A20 conditional knockout mice. The deficiency of A20 in B cells makes them hyperresponsive after an appropriate activation stimuli, and this phenomenon leads to a higher NF-κB activation, as well as an enhanced proliferation and survival (66). Recent results revealed that the level of pro-inflammatory cytokines including IL-6 increases in *Tnfaip3fl/fl CD19-Cre* mice. Interleukin 6 causes an expansion of myeloid and effector T cells as well as a loss of B-cell tolerance (66, 67). The reason may be due to the fact that the deficiency of A20 results in the accumulation of K63-linked ubiquitination by TRAF6 and RIP stimulation, causing prolonged activation of NF-κB signaling pathway and abnormal expression of pro-inflammatory cytokines. A20 also induces RIP2 deubiquitylation, thereby negatively regulating NF-κB activation and inducing the production of pro-inflammatory cytokines by the intracellular PRR and NOD2. Thus, A20 plays a crucial role in B-cell homeostasis and the control of inflammatory responses.

In T-cell development, T lymphocytes express high levels of A20, which decrease after T-cell activation. A great amount of evidence is available regarding the effect of A20 on natural killer T (NKT) cell development. Although A20 is not necessary for the survival of immature NKT cells, it is an important regulator in mediating their maturation. According to the secretion of cytokines, NKT cells are classified into three subgroups, such as NKT1, NKT2, and NKT17, which are characterized by the production of IL-4, IL-17, and interferon γ (IFN-γ). The loss of A20 in T cells can greatly reduce the number of mature NKT cells, but does not affect the early stages of immature NKT cells. In other words, the loss of A20 reduces the quantity of NKT1 and NKT2 cells, without affecting NKT17 cells in organs and the peripheral blood (68, 69).

A20 plays a vital role in mediating CD8 T-cell response, which involves the inhibition of NF-κB signaling pathway. A20 deficiency in mature T cells can lead to excessive production of IL-2 and IFN-γ in CD8^+^ T cells by increasing NF-κB activation. High expression of A20 in tumor-infiltrating CD8^+^ T cells has been reported to be related to poor antitumor immune response, and the loss of A20 is associated with the increased ability of CD8^+^ T cells in tumor clearance. However, another study suggested that the function of A20 in regulating T cells is quite complex, because it could regulate primary and memory responses of CD8^+^ T cells in opposite manners (70, 71). A20 also shows vital influence on the survival of activated CD4^+^ T cells, involving the K5 ubiquitination of RIPK3, which induces the formation of the RIPK1–RIPK3 complex required to induce necrotic cell death. Therefore, A20 deficiency is important for the ubiquitination of RIPK3 and the formation of the RIPK1–RIPK3 complex, which exacerbates the death of CD4^+^ T cells (72). Additionally, A20 mediated CD4^+^ T-cell survival through promoting autophagy, which is caused by the inactivation of mTOR complex 1 (mTORC1), a major inhibitor of autophagy (73, 74).

As regards T-cell tolerance, the regulating mechanism involves inability to induce and regulatory T cell (Treg)–mediated suppression of autoreactive T cells, which were eliminated during central tolerance (75, 76). A20 plays a negative regulatory role in thymus development of Tregs by inhibiting RelA (a classic member of NF-κB). The specific loss of A20 in T cells is related to the increase of Tregs in the thymus tissue and surrounding lymphoid organs. Nevertheless, the unusual thing is that A20 deficiency has no effect on the survival or proliferation of Tregs, which seems to reduce the dependence of thymic Treg precursor cells on IL-2 during development *in vivo* (77).

### CYLD

CYLD was initially identified as a tumor suppressor, and its mutation leads to familial cylindromatosis. The mutation often occurs in the carboxy-terminal (C-terminal) portion of CYLD, which contains a DUB domain 10, interacts with NEMO, and has deubiquitinating activity (78, 79). It is now evident as demonstrated by functional proteomics that CYLD is a member of the USP family of DUBs that negatively regulates NF-κB activation by binding to multiple signaling molecules including NF-κB essential modulator, two IKK regulatory proteins (members of the TRAF family), the NF-κB coactivator BCL-3, the IKK [inhibitor of NF-κB (IκB) kinase], the steroid receptor coactivator (SRC) protein tyrosine kinase LCK, RIP1, and TRPA1 (transient receptor potential channel A1) (79–87).

Recent evidence suggests that CYLD is a protein specifically linked to K63, which facilitates the interaction of CYLD targets in the recruitment and activation of downstream signaling molecules (88, 89). Because of the unique structure of the USP catalytic domain, CYLD has specific determinants that can mediate the uncoupling of K63-linked ubiquitin chains. However, it does not mean that CYLD is precisely a K63-specific DUB, because there is evidence from two studies indicating that CYLD has activity to prevent the proteasomal degradation of some target proteins by targeting toward K48-linked ubiquitin chains (82, 85, 90–92). Moreover, adaptor proteins may be involved in the function of CYLD. For instance, p62, one adaptor protein, can promote the deubiquitylation of TRAF6 (one of p62 targets) by CYLD. Moreover, p62 is able to regulate the DUB activity of CYLD through induction of its ubiquitylation (84, 93).

An important function of CYLD is to regulate the immune response. CYLD deficiency leads to spontaneous B-cell activation and proliferation (94). As regards the regulation of the innate immunity, CYLD activates the nuclear factor of *Streptococcus pneumoniae*–activated T cells by deubiquitinating the upstream kinase TAK1, thus inducing its inhibition (95).

The first DUB shown to regulate thymocyte development is CYLD. The role of CYLD in the regulation of thymocyte development involves IKK activation (96). Moreover, defined by the expression of T4 coreceptors CD4 and CD8, T-cell development in the thymus is divided into three different stages: double-negative, double-positive (DP), and single-positive (SP) cells. The loss of CYLD attenuates thymocyte development because it affects the DP to SP stages, leading to a reduced quantity of T cells in the peripheral lymphoid organs. In regulating T-cell receptor (TCR) signaling during the transition from DP thymocytes to mature SP thymocytes, CYLD targets the tyrosine kinase LCK, playing a critical role in this transition (85). In addition, CYLD regulates the differentiation of myeloid thymocytes required for the negative selection of thymocytes (97). In contrast to A20, CYLD also has an important role in regulating NKT-cell development. CYLD is not only essential for the maturation of NKT cell, but also for the survival of immature NKT cells. Because of abnormal activation of NF-κB, CYLD deficiency attenuates the signal transduction of NKT cells stimulated by IL-7 (98).

In the activation and survival of T cells, CYLD can regulate the dynamic ubiquitination of TAK1 and thus controls TCR/CD28 stimulation in T cells under homeostatic conditions. CYLD deficiency results in the hyperactivation of IKK, JNK, and the downstream transcription factor NF-κB by hyperubiquitination and activation of TAK1 (86). Therefore, CYLD plays an important negative regulator role in TCR activation and homeostasis. Like A20, CYLD negatively regulates the Treg development; thus, CYLD deficiency increases the frequency of Treg in the thymus tissue and peripheral lymphoid organs (99). Because NF-κB is an important inducer in Treg development, CYLD mainly regulates Treg development by inhibiting the NF-κB pathway. Furthermore, CYLD can regulate the development of Treg by inhibiting TGFβ signaling that in turn deubiquitinates smad7 (100, 101). An evidence showed that although CYLD inhibits the development of Tregs, it is regulating the immune suppressive function of Tregs, because enhanced Treg production was observed in mice expressing the CYLD (ex7/8), a non-functional CYLD splice variant (102).

### USP15

In marked contrast with CYLD and A20, USP15 negatively regulates K48-linked ubiquitination of IκBα, which triggers IκBα proteolysis and the nuclear translocation of NF-κB, as well as downstream signaling pathways (103). Recent studies showed that the NFAT signaling is also regulated by USP15 ubiquitin. First, USP15 interacts with MDM2, inhibits ubiquitination, and stabilizes MDM2, an important E3 ligase that mediates the ubiquitination and proteolysis of NFATc2 members of the NFAT family and negatively regulates TCR signals. Subsequently, the activated NFATc2 is conjugated to the K48 ubiquitin chain through the E3 ubiquitin ligase MDM2, inducing its proteasome degradation (56). Because of the ubiquitin-dependent degradation, together with TCR/CD28 stimulation, MDM2 can be transiently down-regulated, and the loss of USP15 greatly promotes the degradation of MDM2 in T cells (56). Therefore, USP15 is deemed as an essential adaptor protein for MDM2-mediated NFAT ubiquitination and T-cell activation.

In T-cell differentiation, USP15 regulates IFN-γ production in activated CD4^+^ T cells at early stage. The main feature of T_H_1 cells is the production of cytokine IFN-γ, as well as the participation in the immune responses against intracellular pathogens (56, 104). The lack of USP15 makes CD4^+^ naive T cells highly responsive to IFN-γ produced by TCR/CD28 stimulation, thus leading to promoted T_H_1 differentiation *in vitro* under the stimulation of a suboptimal dose of T_H_1 polarized cytokine IL-12. In addition, USP15 deficiency in a mouse tumorigenic model enhanced T_H_1 response *in vivo* (56). As mentioned previously, USP15 is not only the DUB of MDM2, but also mediates the ubiquitination of K48 and the degradation of activated NFATc2. NFATc2 is a transcription factor that is critically related to the induction of IFN-γ (56). Some studies show that USP15 is involved in the differentiation of T_H_17 cells. USP15 targets RORγt (T_H_17 lineage transcription factor) for deubiquitylation, but it regulates function rather than the stability of RORγt. The mechanism of action is the following: USP15 increases the association between RORγt and SRC1 by removing ubiquitin from lysine 446 of RORγt, thereby facilitating the transactivation function of RORγt and T_H_17 differentiation (105).

### DUBA

The DUBA is an OTU family member (also called OTUD5), which was found acting as a negative regulator of type I IFN production through siRNA screening (106). Like A20, DUBA has an OTU domain and can selectively cleave K63-linked ubiquitin chains in transfected cells. However, unlike A20 and CYLD, DUBA is not necessary for the negative regulation of NF-κB, because knockdown of DUBA via its targeted specific siRNA shows almost negligible effects on the activation of NF-κB is (106). In contrast, DUBA selectively regulates the activation of IFN-regulatory factor 3 (IRF3) and IRF7, both regulating IFN expression. DUBA not only interacts with TRAF3 and inhibits TRAF3 ubiquitination, but also interrupts the interaction between TRAF3 and TBK1. Therefore, our hypothesis is that the K63-linked ubiquitin chain of TRAF3 may promote its interaction with the TBK1–IKKε complex (107, 108).

Deubiquitylating enzyme A is another T_H_17 regulator. On the one hand, DUBA deletion in T cells promotes the production of T_H_17 cells. On the other hand, DUBA-deficient Tregs, which still have immunosuppressive functions *in vitro* and *in vivo*, can produce IL-17A under TCR stimulation (109). The mechanism involved is that DUBA stabilizes UBR5, which is an E3 ubiquitin ligase that mediates the ubiquitination and proteasomal degradation of RORγt of the T_H_17 lineage. Therefore, DUBA or UBR5 knockout can enhance the level of RORγt and promote T_H_17 cell differentiation (109).

### USP9X

Unlike the several DUBs mentioned previously, which can negatively regulate TCR-stimulated NF-κB signaling, USP9X regulates TCR-proximal signaling and T-cell activation. USP9X binds to Bcl10 and inhibits its ubiquitination under TCR stimulation by deleting the K48-linked ubiquitin chain from Bcl10 (110). A recent study suggests that although USP9X exerts a positive role in TCR signaling, T cell–specific USP9X-deficient mice still have a large number of antigen-stimulated T cells, as well as expanded PD-1– and OX40-expressing populations, which is actually consistent with immune hyperactivity, thus developing a lupus-like autoimmune disease. This effect may be due to a defect in the negative selection of thymocytes in USP9X-deficient mice and consequent generation of self-reactive T cells (111, 112). USP9X also uncouples K48-linked polyubiquitin chains from Themis, a TCR proximal signal molecule that regulates thymocyte development (111). These findings highlight the critical role of USP9X in regulating TCR signaling in thymocytes and peripheral T cells, which also indicates multiple targets are involved in such regulation.

### Perspective

In addition to the well-established classical functions, DUBs also play crucial roles in the immunological regulation of tumors. In a deubiquitination-dependent manner, DUBs not only can stabilize the key immunosuppressive checkpoint PD-L1 to cause the enhancement of tumor-immune escape, but also can directly affect T-cell activation and consequent antitumor immune response by exerting an action on the critical regulators of T-cell activity. Deubiquitinating enzymes act as secondary checkpoints to determine the efficacy of current tumor immunotherapies at the level of posttranslational modification.

Nowadays, small molecule inhibitors targeting DUBs are constantly being developed (Table 2). For instance, WP1130 is designed as a relatively broad range inhibitor to block the activity of USP5, USP9X, USP14, and UCH37. In addition, VLX1570, a USP14 and UCHL5 inhibitor, has entered the first phase of clinical trials of multiple myeloma (NCT02372240). Considered the predominance of immune checkpoint blockade in immunotherapy, as well as the mainstream status of immunotherapy in cancer therapy, DUBs-targeting strategy will have a great translational potential and application prospect in the future cancer immunotherapy. Therefore, it is urgent to further identify the core DUBs in tumor immune regulation and clarify the target and mechanism of its action in depth.

**Table 2:** Deubiquitinating enzyme–targeted drug candidates.

**Name**	**Target(s)**	**Efficacy**	**References**
PR-619	ATXN3, BAP1, JOSD2, OTUD5, UCH-L1, UCH-L3, UCH-L5/UCH37, USP1, 2, 4, 5, 7, 8, 9X, 10, 14, 15, 16, 19, 20, 22, 24, 28, 47, 48, VCIP135, YOD1, PLpro, DEN1, SENP6	EC50: 1–20 μM	(113)
P5091 (P005091)	USP7, 47	EC50: 4.2 μM, 4.3 μM	(114, 115)
P22077	USP7, 47	EC50: 8 μM	(113, 116)
HBX41108	USP7	IC50: 0.27 μM	(117)
HBX19818	USP7	IC50: 28.1 μM	(118)
HBX28258	USP7	IC50: 22.6 μM	(118)
9-oxo-9H-indeno [1,2-b]pyrazine-2,3-dicarbonitrile	USP7, USP8	IC50: 3.5 μM, 0.29 μM	(119)
b-AP15	UCHL5	IC50: 2.1 μM	(120)
VLX1570	USP14, UCHL5	EC50: 29 nM	(121)
Degrasyn (WP1130)	USP5, USP9X, USP14, UCH37	IC50: 1-5 μM	(122, 123)
IU1	USP14	IC50: 4.7 μM	(124)
pimozide	USP1/UAF1	IC50: 2 μM	(125)
GW7647	USP1/UAF1	IC50: 5 μM	(125)
Isatin O-acyl oxime derivatives (30, 50, 51)	UCHL1, UCHL3	IC50: 0.80-0.94 μM, 17-25 μM	(126)
AZ1	USP25, USP28	IC50: 0.62 μM, 0.7 μM	(127)
ML364	USP2, USP8	IC50: 1.1 μM, 0.95 μM	(128)
ML323	USP1-UAF1	IC50: 76 nM	(129)
TCID	UCH-L3	IC50: 0.6 μM	(126)
Vialinin A	USP4, USP5	IC50: 1–25 μM	(130)
XL188	USP7	IC50: 90–190 nM	(131)
Chalcone derivatives (AM146, RA-9, RA-14)	DUB	IC50: 1-13 μM	(132)
GRL0617	PLpro	EC50: 10–15 μM	(133)

## Author Contributions

XH, TL, and XB conceived the project and supervised the drafting process. XH and XZ drafted and revised the manuscript with the assistance of JX. XW, GZ, TT, and XS discussed and commented on the manuscript. All authors contributed to the article and approved the submitted version.

## Conflict of Interest

The authors declare that the research was conducted in the absence of any commercial or financial relationships that could be construed as a potential conflict of interest.

## Abbreviations

DUB: deubiquitinating enzyme
E1: ubiquitin-activating enzyme
E2: ubiquitin-binding enzyme
E3: ubiquitin ligase
USP: ubiquitin-specific protease
UCH: ubiquitin COOH terminal hydrolase
MJD: Machado-Josephine-containing protease
OUT: ovarian tumor protease
MINDY: motif that interacts with the novel DUB family containing ubiquitin
JAMM: JAB1/MPN/MOV34 metalloprotease DUB
SENP: Sentrin/ SUMO-specific protease
DeSI: de-SUMO-glycosylated isopeptidase
NEDP1: NEDD8-specific protease 1
PTM: posttranslational modification
HCC: hepatocellular carcinoma
CSN5: COP9 signalosome 5
JAMM: JAB1/MPN/Mov34 metalloenzyme
CCL5: C-C motif chemokine ligand 5
TNF: tumor necrosis factor
TNFR: TNF receptor
TLR: Toll-like receptor
NOD2: nucleotide-binding oligomerization domain protein 2
N-terminus: amino-terminus
OUT: ovarian tumor–related proteases
RIPK3: receptor-interacting protein 3
mTORC1: mTOR complex 1
IκB: inhibitor of NF-κB
IKK: IκB kinase
TRPA1: transient receptor potential channel A1
DN: double negative
DP: double positive
SP: single positive
mTEC: myeloid thymocyte
NKT: natural killer T
SRC1: steroid receptor coactivator 1
DUBA: deubiquitylating enzyme A
IFN: interferon
IRF3: IFN-regulatory factor 3.

## References

[1] Deshaies RJ Joazeiro CAP. RING domain E3 ubiquitin ligases. Annu Rev Biochem. (2009) 78:399–434. 10.1146/annurev.biochem.78.101807.093809 19489725

[2] Komander D Rape M. The ubiquitin code. Annu Rev Biochem. (2012) 81:203–29. 10.1146/annurev-biochem-060310-170328 22524316

[3] Yau R Rape M. The increasing complexity of the ubiquitin code. Nat Cell Biol. (2016) 18:579–86. 10.1038/ncb3358 27230526

[4] Swatek KN Komander D. Ubiquitin modifications. Cell Res. (2016) 26:399–422. 10.1038/cr.2016.39 27012465 PMC4822133

[5] Kim W Bennett Eric J Huttlin Edward L Guo A Li J Possemato A. Systematic and quantitative assessment of the ubiquitin-modified proteome. Mol Cell. (2011) 44:325–40. 10.1016/j.molcel.2011.08.025 21906983 PMC3200427

[6] Schulman B Harper J. Ubiquitin-like protein activation by E1 enzymes: the apex for downstream signalling pathways. Nat Rev Mol Cell Biol. (2009) 10:319–31. 10.1038/nrm2673 19352404 PMC2712597

[7] Ye Y Rape M. Building ubiquitin chains: E2 enzymes at work. Nat Rev Mol Cell Biol. (2009) 10:755–64. 10.1038/nrm2780 19851334 PMC3107738

[8] Buetow L Huang DT. Structural insights into the catalysis and regulation of E3 ubiquitin ligases. Nat Rev Mol Cell Biol. (2016) 17:626–42. 10.1038/nrm.2016.91 27485899 PMC6211636

[9] Reyes-Turcu FE Ventii KH Wilkinson KD. Regulation and cellular roles of ubiquitin-specific deubiquitinating enzymes. Annu Rev Biochem. (2009) 78:363–97. 10.1146/annurev.biochem.78.082307.091526 19489724 PMC2734102

[10] Wilkinson KD Tashayev VL O'Connor LB Larsen CN Kasperek E Pickart CM. Metabolism of the polyubiquitin degradation signal: structure, mechanism, and role of isopeptidase T. Biochemistry. (1995) 34:14535–46. 10.1021/bi00044a032 7578059

[11] Komander D Clague M Urbé S. Breaking the chains: structure and function of the deubiquitinases. Nat Rev Mol Cell Biol. (2009) 10:550–63. 10.1038/nrm2731 19626045

[12] Abdul rehman Sa Kristariyanto Y Choi S-Y Nkosi P Weidlich S Labib K. MINDY-1 is a member of an evolutionarily conserved and structurally distinct new family of deubiquitinating enzymes. Molecular Cell. (2016) 63146–55. 10.1016/j.molcel.2016.05.009 27292798 PMC4942677

[13] Hickey CM Wilson NR Hochstrasser M. Function and regulation of SUMO proteases. Nat Rev Mol Cell Biol. (2012) 13:755–66. 10.1038/nrm3478 23175280 PMC3668692

[14] Shin EJ Shin HM Nam E Kim WS Kim JH Oh BH. DeSUMOylating isopeptidase: a second class of SUMO protease. Embo Rep. (2012) 13:339–46. 10.1038/embor.2012.3 22370726 PMC3321169

[15] Fernandez DJ Hess S Knobeloch KP. Strategies to target ISG15 and USP18 toward therapeutic applications. Front Chem. (2020) 7:923. 10.3389/fchem.2019.00923 32039148 PMC6985271

[16] Ronau JA Beckmann JF Hochstrasser M. Substrate specificity of the ubiquitin and Ubl proteases. Cell Res. (2016) 26:441–56. 10.1038/cr.2016.38 27012468 PMC4822132

[17] Mevissen TET Komander D. Mechanisms of deubiquitinase specificity and regulation. Annu Rev Biochem. (2017) 86:159–92. 10.1146/annurev-biochem-061516-044916 28498721

[18] Sahtoe DD Sixma TK. Layers of DUB regulation. Trends Biochem Sci. (2015) 40:456–67. 10.1016/j.tibs.2015.05.002 26073511

[19] Sharma P Allison JP. The future of immune checkpoint therapy. Science. (2015) 348:56–61. 10.1126/science.aaa8172 25838373

[20] Pardoll DM. The blockade of immune checkpoints in cancer immunotherapy. Nat Rev Cancer. (2012) 12:252–64. 10.1038/nrc3239 22437870 PMC4856023

[21] Brahmer JR Tykodi SS Chow LQM Hwu WJ Topalian SL Hwu P. Safety and activity of anti-PD-L1 antibody in patients with advanced cancer. N Engl J Med. (2012) 366:2455–65. 10.1056/NEJMoa1200694 22658128 PMC3563263

[22] Hsu JM Li CW Lai YJ Hung MC. Posttranslational modifications of PD-L1 and their applications in cancer therapy. Cancer Res. (2018) 78:6349–53. 10.1158/0008-5472.CAN-18-1892 30442814 PMC6242346

[23] Lee HJ Kim MS Shin JM Park TJ Chung HM Baek KH. The expression patterns of deubiquitinating enzymes, USP22 and Usp22. Gene Exp Patterns. (2006) 6:277–84. 10.1016/j.modgep.2005.07.007 16378762

[24] Zhang XY Varthi M Sykes SM Phillips C Warzecha C Zhu W. The putative cancer stem cell marker USP22 is a subunit of the human SAGA complex required for activated transcription and cell-cycle progression. Mol Cell. (2008) 29:102–11. 10.1016/j.molcel.2007.12.015 18206973 PMC2254522

[25] Liang JX Ning Z Gao W Ling J Wang AM Luo HF. Ubiquitin-specific protease 22-induced autophagy is correlated with poor prognosis of pancreatic cancer. Oncol Rep. (2014) 32:2726–34. 10.3892/or.2014.3508 25241857

[26] Ning Z Wang AM Liang JX Xie YP Liu JW Feng L. USP22 promotes the G1/S phase transition by upregulating FoxM1 expression via beta-catenin nuclear localization and is associated with poor prognosis in stage II pancreatic ductal adenocarcinoma. Int J Oncol. (2014) 45:1594–608. 10.3892/ijo.2014.2531 24993031

[27] Schrecengost RS Dean JL Goodwin JF Schiewer MJ Urban MW Stanek TJ. USP22 Regulates oncogenic signaling pathways to drive lethal cancer progression. Cancer Res. (2014) 74:272–86. 10.1158/0008-5472.CAN-13-1954 24197134 PMC3947329

[28] Liu YL Yang YM Xu H Dong XS. Increased expression of ubiquitin-specific protease 22 can promote cancer progression and predict therapy failure in human colorectal cancer. J Gastroenterol Hepatol. (2010) 25:1800–5. 10.1111/j.1440-1746.2010.06352.x 21039844

[29] Huang X Zhang Q Lou Y Wang J Zhao X Wang L. USP22 deubiquitinates CD274 to suppress anti-cancer immunity. Cancer Immunol Res. (2019) 7:1580–90. 10.1158/2326-6066.CIR-18-0910 31399419

[30] Huang X Zhang X Bai X Liang T. Blocking PD-L1 for anti-liver cancer immunity: USP22 represents a critical cotarget. Cell Mol Immunol. (2019) 17:677–9. 10.1038/s41423-019-0348-4 31857703 PMC7331717

[31] Li J Yuan S Norgard RJ Yan F Yamazoe T Blanco A. Tumor cell-intrinsic USP22 suppresses antitumor immunity in pancreatic cancer. Cancer Immunol Res. (2019) 8:282–91. 10.1158/2326-6066.CIR-19-0661 31871120 PMC7173406

[32] Chen CL Pan QZ Zhao JJ Wang Y Li YQ Wang QJ. PD-L1 expression as a predictive biomarker for cytokine-induced killer cell immunotherapy in patients with hepatocellular carcinoma. Oncoimmunology. (2016) 5:e1176653. 10.1080/2162402X.2016.1176653 27622026 PMC5006896

[33] Kamath SD Kalyan A Kircher S Nimeiri H Fought AJ Benson A. Ipilimumab and gemcitabine for advanced pancreatic cancer: a phase IB study. Oncologist. (2019) 25:e808–e15. 10.1634/theoncologist.2019-0473 31740568 PMC7216436

[34] Wu Y Deng J Rychahou PG Qiu SM Evers BM Zhou BPH. Stabilization of snail by NF-kappa B is required for inflammation-induced cell migration and invasion. Cancer Cell. (2009) 15:416–28. 10.1016/j.ccr.2009.03.016 19411070 PMC2881229

[35] Liu YL Shah SV Xiang XY Wang JH Deng ZB Liu CR. COP9-Associated CSN5 regulates exosomal protein deubiquitination and sorting. Am J Pathol. (2009) 174:1415–25. 10.2353/ajpath.2009.080861 19246649 PMC2671372

[36] Kotiguda GG Weinberg D Dessau M Salvi C Serino G Chamovitz DA. The organization of a CSN5-containing subcomplex of the COP9 signalosome. J Biol Chem. (2012) 287:42031–41. 10.1074/jbc.M112.387977 23086934 PMC3516749

[37] Cope GA Suh GSB Aravind L Schwarz SE Zipursky SL Koonin EV. Role of predicted metalloprotease motif of Jab1/Csn5 in cleavage of Nedd8 from Cul1. Science. (2002) 298:608–11. 10.1126/science.1075901 12183637

[38] Lim SO Li CW Xia W Cha JH Chan LC Wu Y. Deubiquitination and stabilization of PD-L1 by CSN5. Cancer Cell. (2016) 30:925–39. 10.1016/j.ccell.2016.10.010 27866850 PMC5171205

[39] Liu C Yao Z Wang J Zhang W Yang Y Zhang Y. Macrophage-derived CCL5 facilitates immune escape of colorectal cancer cells via the p65/STAT3-CSN5-PD-L1 pathway. Cell Death Differ. (2019) 27:1765–81. 10.1038/s41418-019-0460-0 31802034 PMC7244707

[40] Kapuria V Peterson LF Fang D Bornmann WG Talpaz M Donato NJ. Deubiquitinase inhibition by small-molecule WP1130 triggers aggresome formation and tumor cell apoptosis. Cancer Res. (2010) 70:9265–76. 10.1158/0008-5472.CAN-10-1530 21045142

[41] Schwickart M Huang XD Lill JR Liu JF Ferrando R French DM. Deubiquitinase USP9X stabilizes MCL1 and promotes tumour cell survival. Nature. (2010) 463:103–14. 10.1038/nature08646 20023629

[42] McGarry E Gaboriau D Rainey MD Restuccia U Bachi A Santocanale C. The deubiquitinase USP9X maintains DNA replication fork stability and DNA damage checkpoint responses by regulating CLASPIN during S-Phase. Cancer Res. (2016) 76:2384–93. 10.1158/0008-5472.CAN-15-2890 26921344

[43] Wolfsperger F Hogh-Binder SA Schittenhelm J Psaras T Ritter V Bornes L. Deubiquitylating enzyme USP9x regulates radiosensitivity in glioblastoma cells by Mcl-1-dependent and -independent mechanisms. Cell Death Dis. (2016) 7:e2039. 10.1038/cddis.2015.405 26775694 PMC4816183

[44] Wu JJ Guo WZ Wen DH Hou GY Zhou AP Wu WJ. Deubiquitination and stabilization of programmed cell death ligand 1 by ubiquitin-specific peptidase 9, X-linked in oral squamous cell carcinoma. Cancer Med. (2018) 7:4004–11. 10.1002/cam4.1675 29992764 PMC6089178

[45] Vlasschaert C Xia XH Coulombe J Gray DA. Evolution of the highly networked deubiquitinating enzymes USP4, USP15, and USP11. BMC Evol Biol. (2015) 15:230. 10.1186/s12862-015-0511-1 26503449 PMC4624187

[46] Sun WJ Tan XJ Shi Y Xu GF Mao RF Gu X. USP11 negatively regulates TNF alpha-induced NF-kappa B activation by targeting on I kappa B alpha. Cell Signal. (2010) 22:386–94. 10.1016/j.cellsig.2009.10.008 19874889 PMC2794974

[47] Zhong B Liu X Wang X Liu X Li H Darnay BG. Ubiquitin-specific protease 25 regulates TLR4-dependent innate immune responses through deubiquitination of the adaptor protein TRAF3. Sci Signal. (2013) 6:ra35. 10.1126/scisignal.2003708 23674823 PMC5763475

[48] Zhao MM Song K Hao WZ Wang LY Patil G Li QM. Non-proteolytic ubiquitination of OTULIN regulates NF-kappa B signaling pathway. J Mol Cell Biol. (2020) 12:163–75. 10.1093/jmcb/mjz081 31504727 PMC7181720

[49] Hu H Wang H Xiao Y Jin J Chang JH Zou Q. Otud7b facilitates T cell activation and inflammatory responses by regulating Zap70 ubiquitination. J Exp Med. (2016) 213:399–414. 10.1084/jem.20151426 26903241 PMC4813674

[50] Wang F Wang LQ Wu J Sokirniy I Nguyen P Bregnard T. Active site-targeted covalent irreversible inhibitors of USP7 impair the functions of Foxp3+T-regulatory cells by promoting ubiquitination of Tip60. PLos ONE. (2017) 12:e0189744. 10.1371/journal.pone.0189744 29236775 PMC5728538

[51] Fu C Zhu X Xu P Li Y. Pharmacological inhibition of USP7 promotes antitumor immunity and contributes to colon cancer therapy. Oncotargets Ther. (2019) 12:609–16. 10.2147/OTT.S182806 30697058 PMC6339463

[52] Zhang YW Luo YQ Wang YN Liu H Yang Y Wang Q. Effect of deubiquitinase USP8 on hypoxia/reoxygenation-induced inflammation by deubiquitination of TAK1 in renal tubular epithelial cells. Int J Mol Med. (2018) 42:3467–76. 10.3892/ijmm.2018.3881 30221684

[53] Palazon-Riquelme P Worboys JD Green J Valera A Martin-Sanchez F Pellegrini C. USP7 and USP47 deubiquitinases regulate NLRP3 inflammasome activation. Embo Rep. (2018) 19:e44766. 10.15252/embr.201744766 30206189 PMC6172458

[54] Py BF Kim MS Vakifahmetoglu-Norberg H Yuan JY. Deubiquitination of NLRP3 by BRCC3 critically regulates inflammasome activity. Mol Cell. (2013) 49:331–8. 10.1016/j.molcel.2012.11.009 23246432

[55] Zhang XN Berger FG Yang JH Lu XB. USP4 inhibits p53 through deubiquitinating and stabilizing ARF-BP1. Embo J. (2011) 30:2177–89. 10.1038/emboj.2011.125 21522127 PMC3117646

[56] Zou Q Jin J Hu HB Li HYS Romano S Xiao YC. USP15 stabilizes MDM2 to mediate cancer-cell survival and inhibit antitumor T cell responses. Nat Immunol. (2014) 15:562–70. 10.1038/ni.2885 24777531 PMC4032322

[57] Han L Yang J Wang X Wu Q Yin S Li Z. The E3 deubiquitinase USP17 is a positive regulator of retinoic acid-related orphan nuclear receptor γt (RORγt) in Th17 cells. J Biol Chem. (2014) 289:25546–55. 10.1074/jbc.M114.565291 25070893 PMC4162160

[58] Liu X Li H Zhong B Blonska M Gorjestani S Yan M. USP18 inhibits NF-κB and NFAT activation during Th17 differentiation by deubiquitinating the TAK1-TAB1 complex. J Exp Med. (2013) 210:1575–90. 10.1084/jem.20122327 23825189 PMC3727316

[59] Wertz IE O'Rourke KM Zhou HL Eby M Aravind L Seshagiri S. De-ubiquitination and ubiquitin ligase domains of A20 downregulate NF-kappa B signalling. Nature. (2004) 430:694–99. 10.1038/nature02794 15258597

[60] Lee EG Boone DL Chai S Libby SL Chien M Lodolce JP. Failure to regulate TNF-induced NF-kappaB and cell death responses in A20-deficient mice. Science. (2000) 289:2350–54. 10.1126/science.289.5488.2350 11009421 PMC3582399

[61] Hitotsumatsu O Ahmad RC Tavares R Wang M Philpott D Turer EE. The ubiquitin-editing enzyme A20 restricts nucleotide-binding oligomerization domain containing 2-triggered signals. Immunity. (2008) 28:381–90. 10.1016/j.immuni.2008.02.002 18342009 PMC3606373

[62] Boone DL Turer EE Lee EG Ahmad RC Wheeler MT Tsui C. The ubiquitin-modifying enzyme A20 is required for termination of Toll-like receptor responses. Nat Immunol. (2004) 5:1052–60. 10.1038/ni1110 15334086

[63] Nijman SM Luna-Vargas MP Velds A Brummelkamp TR Dirac AM Sixma TK. A genomic and functional inventory of deubiquitinating enzymes. Cell. (2005) 123:773–86. 10.1016/j.cell.2005.11.007 16325574

[64] Shembade N Ma A Harhaj EW. Inhibition of NF-kappaB signaling by A20 through disruption of ubiquitin enzyme complexes. Science. (2010) 327:1135–9. 10.1126/science.1182364 20185725 PMC3025292

[65] Turer EE Tavares RM Mortier E Hitotsumatsu O Advincula R Lee B. Homeostatic MyD88-dependent signals cause lethal inflamMation in the absence of A20. J Exp Med. (2008) 205:451–64. 10.1084/jem.20071108 18268035 PMC2271029

[66] Tavares RM Turer EE Liu CL Advincula R Scapini P Rhee L. The ubiquitin modifying enzyme A20 restricts B cell survival and prevents autoimmunity. Immunity. (2010) 33:181–91. 10.1016/j.immuni.2010.07.017 20705491 PMC2931361

[67] Chu Y Vahl JC Kumar D Heger K Bertossi A Wojtowicz E. B cells lacking the tumor suppressor TNFAIP3/A20 display impaired differentiation and hyperactivation and cause inflammation and autoimmunity in aged mice. Blood. (2011) 117:2227–36. 10.1182/blood-2010-09-306019 21088135

[68] Drennan MB Govindarajan S Verheugen E Coquet JM Staal J McGuire C. NKT sublineage specification and survival requires the ubiquitin-modifying enzyme TNFAIP3/A20. J Exp Med. (2016) 213:1973–81. 10.1084/jem.20151065 27551157 PMC5030796

[69] Lee YJ Holzapfel KL Zhu JF Jameson SC Hogquist KA. Steady-state production of IL-4 modulates immunity in mouse strains and is determined by lineage diversity of iNKT cells. Nat Immunol. (2013) 14:1146–1126. 10.1038/ni.2731 24097110 PMC3824254

[70] Giordano M Roncagalli R Bourdely P Chasson L Buferne M Yamasaki S. The tumor necrosis factor alpha-induced protein 3 (TNFAIP3, A20) imposes a brake on antitumor activity of CD8 T cells. Proc Natl Acad Sci USA. (2014) 111:11115–20. 10.1073/pnas.1406259111 25024217 PMC4121810

[71] Just S Nishanth G Buchbinder JH Wang X Naumann M Lavrik I. A20 curtails primary but augments secondary CD8^+^ T cell responses in intracellular bacterial infection. Sci Rep. (2016) 6:39796. 10.1038/srep39796 28004776 PMC5177869

[72] Onizawa M Oshima S Schulze-Topphoff U Oses-Prieto JA Lu T Tavares R. The ubiquitin-modifying enzyme A20 restricts ubiquitination of the kinase RIPK3 and protects cells from necroptosis. Nat Immunol. (2015) 16:618–27. 10.1038/ni.3172 25939025 PMC4439357

[73] Matsuzawa Y Oshima S Takahara M Maeyashiki C Nemoto Y Kobayashi M. TNFAIP3 promotes survival of CD4 T cells by restricting MTOR and promoting autophagy. Autophagy. (2015) 11:1052–62. 10.1080/15548627.2015.1055439 26043155 PMC4590588

[74] Linares JF Duran A Yajima T Pasparakis M Moscat J Diaz-Meco MT. K63 polyubiquitination and activation of mTOR by the p62-TRAF6 complex in nutrient-activated cells. Mol Cell. (2013) 51:283–96. 10.1016/j.molcel.2013.06.020 23911927 PMC3971544

[75] Xing Y Hogquist KA. T-cell tolerance: central and peripheral. Cold Spring Harb Perspect Biol. (2012) 4:e44766. 10.1101/cshperspect.a006957 22661634 PMC3367546

[76] Sakaguchi S Yamaguchi T Nomura T Ono M. Regulatory T cells and immune tolerance. Cell. (2008) 133:775–87. 10.1016/j.cell.2008.05.009 18510923

[77] Fischer JC Otten V Kober M Drees C Rosenbaum M Schmickl M. A20 restrains thymic regulatory T cell development. J Immunol. (2017) 199:2356–65. 10.4049/jimmunol.1602102 28842469 PMC5617121

[78] Bignell GR Warren W Seal S Takahashi M Rapley E Barfoot R. Identification of the familial cylindromatosis tumour-suppressor gene. Nat Genet. (2000) 25:160–65. 10.1038/76006 10835629

[79] Kovalenko A Chable-Bessia C Cantarella G Israel A Wallach D Courtois G. The tumour suppressor CYLD negatively regulates NF-kappa B signalling by deubiquitination. Nature. (2003) 424:801–5. 10.1038/nature01802 12917691

[80] Borodovsky A Ovaa H Kolli N Gan-Erdene T Wilkinson KD Ploegh HL. Chemistry-based functional proteomics reveals novel members of the deubiquitinating enzyme family. Chem Biol. (2002) 9:1149–59. 10.1016/S1074-5521(02)00248-X 12401499

[81] Brummelkamp TR Nijman SM Dirac AM Bernards R. Loss of the cylindromatosis tumour suppressor inhibits apoptosis by activating NF-kappaB. Nature. (2003) 424:797–801. 10.1038/nature01811 12917690

[82] Trompouki E Hatzivassiliou E Tsichritzis T Farmer H Ashworth A Mosialos G. CYLD is a deubiquitinating enzyme that negatively regulates NF-kappaB activation by TNFR family members. Nature. (2003) 424:793–6. 10.1038/nature01803 12917689

[83] Yoshida H Jono H Kai H Li JD. The tumor suppressor cylindromatosis (CYLD) acts as a negative regulator for toll-like receptor 2 signaling via negative cross-talk with TRAF6 AND TRAF7. J Biol Chem. (2005) 280:41111–21. 10.1074/jbc.M509526200 16230348

[84] Jin W Chang M Paul EM Babu G Lee AJ Reiley W. Deubiquitinating enzyme CYLD negatively regulates RANK signaling and osteoclastogenesis in mice. J Clin Invest. (2008) 118:1858–66. 10.1172/JCI34257 18382763 PMC2276399

[85] Reiley WW Zhang M Jin W Losiewicz M Donohue KB Norbury CC. Regulation of T cell development by the deubiquitinating enzyme CYLD. Nat Immunol. (2006) 7:411–17. 10.1038/ni1315 16501569

[86] Reiley WW Jin W Lee AJ Wright A Wu X Tewalt EF. Deubiquitinating enzyme CYLD negatively regulates the ubiquitin-dependent kinase Tak1 and prevents abnormal T cell responses. J Exp Med. (2007) 204:1475–85. 10.1084/jem.20062694 17548520 PMC2118606

[87] Wright A Reiley WW Chang M Jin W Lee AJ Zhang M. Regulation of early wave of germ cell apoptosis and spermatogenesis by deubiquitinating enzyme CYLD. Dev Cell. (2007) 13:705–16. 10.1016/j.devcel.2007.09.007 17981138

[88] Adhikari A Xu M Chen ZJ. Ubiquitin-mediated activation of TAK1 and IKK. Oncogene. (2007) 26:3214–26. 10.1038/sj.onc.1210413 17496917

[89] Massoumi R Chmielarska K Hennecke K Pfeifer A Fassler R. Cyld inhibits tumor cell proliferation by blocking Bcl-3-dependent NF-kappaB signaling. Cell. (2006) 125:665–77. 10.1016/j.cell.2006.03.041 16713561

[90] Stokes A Wakano C Koblan-Huberson M Adra CN Fleig A Turner H. TRPA1 is a substrate for de-ubiquitination by the tumor suppressor CYLD. Cell Signal. (2006) 18:1584–94. 10.1016/j.cellsig.2005.12.009 16500080

[91] Komander D Lord CJ Scheel H Swift S Hofmann K Ashworth A. The structure of the CYLD USP domain explains its specificity for Lys63-linked polyubiquitin and reveals a B box module. Mol Cell. (2008) 29:451–64. 10.1016/j.molcel.2007.12.018 18313383

[92] Xue L Igaki T Kuranaga E Kanda H Miura M Xu T. Tumor suppressor CYLD regulates JNK-induced cell death in drosophila. Dev Cell. (2007) 13:446–54. 10.1016/j.devcel.2007.07.012 17765686

[93] Wooten MW Geetha T Babu JR Seibenhener ML Peng J Cox N. Essential role of sequestosome 1/p62 in regulating accumulation of Lys63-ubiquitinated proteins. J Biol Chem. (2008) 283:6783–89. 10.1074/jbc.M709496200 18174161

[94] Jin W Reiley WR Lee AJ Wright A Wu X Zhang M. Deubiquitinating enzyme CYLD regulates the peripheral development and naive phenotype maintenance of B cells. J Biol Chem. (2007) 282:15884–93. 10.1074/jbc.M609952200 17392286

[95] Lim JH Ha UH Woo CH Xu H Li JD. CYLD is a crucial negative regulator of innate immune response in *Escherichia coli* pneumonia. Cell Microbiol. (2008) 10:2247–56. 10.1111/j.1462-5822.2008.01204.x 18643924

[96] Tsagaratou A Trompouki E Grammenoudi S Kontoyiannis DL Mosialos G. Thymocyte-specific truncation of the deubiquitinating domain of CYLD impairs positive selection in a NF-kappaB essential modulator-dependent manner. J Immunol. (2010) 185:2032–43. 10.4049/jimmunol.0903919 20644164

[97] Reissig S Hovelmeyer N Tang Y Weih D Nikolaev A Riemann M. The deubiquitinating enzyme CYLD regulates the differentiation and maturation of thymic medullary epithelial cells. Immunol Cell Biol. (2015) 93:558–66. 10.1038/icb.2014.122 25601276

[98] Lee AJ Zhou X Chang M Hunzeker J Bonneau RH Zhou D. Regulation of natural killer T-cell development by deubiquitinase CYLD. Embo J. (2010) 29:1600–12. 10.1038/emboj.2010.31 20224552 PMC2876956

[99] Lee AJ Wu X Cheng H Zhou X Cheng X Sun SC. CARMA1 regulation of regulatory T cell development involves modulation of interleukin-2 receptor signaling. J Biol Chem. (2010) 285:15696–703. 10.1074/jbc.M109.095190 20233721 PMC2871435

[100] Oh H Ghosh S. NF-kappaB: roles and regulation in different CD4^+^ T-cell subsets. Immunol Rev. (2013) 252:41–51. 10.1111/imr.12033 23405894 PMC3576882

[101] Zhao Y Thornton AM Kinney MC Ma CA Spinner JJ Fuss IJ. The deubiquitinase CYLD targets Smad7 protein to regulate transforming growth factor beta (TGF-beta) signaling and the development of regulatory T cells. J Biol Chem. (2011) 286:40520–30. 10.1074/jbc.M111.292961 21931165 PMC3220473

[102] Reissig S Hovelmeyer N Weigmann B Nikolaev A Kalt B Wunderlich TF. The tumor suppressor CYLD controls the function of murine regulatory T cells. J Immunol. (2012) 189:4770–6. 10.4049/jimmunol.1201993 23066153

[103] Chen ZJ Parent L Maniatis T. Site-specific phosphorylation of IkappaBalpha by a novel ubiquitination-dependent protein kinase activity. Cell. (1996) 84:853–62. 10.1016/S0092-8674(00)81064-8 8601309

[104] Yamane H Paul WE. Early signaling events that underlie fate decisions of naive CD4^+^ T cells toward distinct T-helper cell subsets. Immunol Rev. (2013) 252:12–23. 10.1111/imr.12032 23405892 PMC3578301

[105] He Z Wang F Ma J Sen S Zhang J Gwack Y. Ubiquitination of RORgammat at Lysine 446 limits Th17 differentiation by controlling coactivator recruitment. J Immunol. (2016) 197:1148–58. 10.4049/jimmunol.1600548 27430721 PMC4976012

[106] Kayagaki N Phung Q Chan S Chaudhari R Quan C O'Rourke KM. DUBA: a deubiquitinase that regulates type I interferon production. Science. (2007) 318:1628–32. 10.1126/science.1145918 17991829

[107] Guo B Cheng G. Modulation of the interferon antiviral response by the TBK1/IKKi adaptor protein TANK. J Biol Chem. (2007) 282:11817–26. 10.1074/jbc.M700017200 17327220

[108] Oganesyan G Saha SK Guo B He JQ Shahangian A Zarnegar B. Critical role of TRAF3 in the Toll-like receptor-dependent and -independent antiviral response. Nature. (2006) 439:208–11. 10.1038/nature04374 16306936

[109] Rutz S Kayagaki N Phung QT Eidenschenk C Noubade R Wang X. Deubiquitinase DUBA is a post-translational brake on interleukin-17 production in T cells. Nature. (2015) 518:417–21. 10.1038/nature13979 25470037

[110] Park Y Jin HS Liu YC. Regulation of T cell function by the ubiquitin-specific protease USP9X via modulating the Carma1-Bcl10-Malt1 complex. Proc Natl Acad Sci USA. (2013) 110:9433–8. 10.1073/pnas.1221925110 23690623 PMC3677447

[111] Garreau A Blaize G Argenty J Rouquie N Tourdes A Wood SA. Grb2-Mediated recruitment of USP9X to LAT enhances themis stability following thymic selection. J Immunol. (2017) 199:2758–66. 10.4049/jimmunol.1700566 28877990

[112] Naik E Webster JD DeVoss J Liu JF Suriben R Dixit VM. Regulation of proximal T cell receptor signaling and tolerance induction by deubiquitinase Usp9X. J Exp Med. (2014) 211:1947–55. 10.1084/jem.20140860 25200027 PMC4172213

[113] Altun M Kramer HB Willems LI McDermott JL Leach CA Goldenberg SJ. Activity-based chemical proteomics accelerates inhibitor development for deubiquitylating enzymes. Chem Biol. (2011) 18:1401–12. 10.1016/j.chembiol.2011.08.018 22118674

[114] Chauhan D Tian Z Nicholson B Kumar KG Zhou B Carrasco R. A small molecule inhibitor of ubiquitin-specific protease-7 induces apoptosis in multiple myeloma cells and overcomes bortezomib resistance. Cancer Cell. (2012) 22:345–58. 10.1016/j.ccr.2012.08.007 22975377 PMC3478134

[115] Weinstock J Wu J Cao P Kingsbury WD McDermott JL Kodrasov MP. Selective dual inhibitors of the cancer-related deubiquitylating proteases USP7 and USP47. ACS Med Chem Lett. (2012) 3:789–92. 10.1021/ml200276j 24900381 PMC4025646

[116] Tian X Isamiddinova NS Peroutka RJ Goldenberg SJ Mattern MR Nicholson B. Characterization of selective ubiquitin and ubiquitin-like protease inhibitors using a fluorescence-based multiplex assay format. Assay Drug Dev Technol. (2011) 9:165–73. 10.1089/adt.2010.0317 21133675 PMC3065724

[117] Colland F Formstecher E Jacq X Reverdy C Planquette C Conrath S. Small-molecule inhibitor of USP7/HAUSP ubiquitin protease stabilizes and activates p53 in cells. Mol Cancer Ther. (2009) 8:2286–95. 10.1158/1535-7163.MCT-09-0097 19671755

[118] Reverdy C Conrath S Lopez R Planquette C Atmanene C Collura V. Discovery of specific inhibitors of human USP7/HAUSP deubiquitinating enzyme. Chem Biol. (2012) 19:467–77. 10.1016/j.chembiol.2012.02.007 22520753

[119] Colombo M Vallese S Peretto I Jacq X Rain JC Colland F. Synthesis and biological evaluation of 9-oxo-9H-indeno[1,2-b]pyrazine-2,3-dicarbonitrile analogues as potential inhibitors of deubiquitinating enzymes. ChemMedChem. (2010) 5:552–8. 10.1002/cmdc.200900409 20186914

[120] D'Arcy P Brnjic S Olofsson MH Fryknäs M Lindsten K De Cesare M. Inhibition of proteasome deubiquitinating activity as a new cancer therapy. Nat Med. (2011) 17:1636–40. 10.1038/nm.2536 22057347

[121] Paulus A Akhtar S Caulfield TR Samuel K Yousaf H Bashir Y. Coinhibition of the deubiquitinating enzymes, USP14 and UCHL5, with VLX1570 is lethal to ibrutinib- or bortezomib-resistant Waldenstrom macroglobulinemia tumor cells. Blood Cancer J. (2016) 6:e492. 10.1038/bcj.2016.93 27813535 PMC5148058

[122] Bartholomeusz GA Talpaz M Kapuria V Kong LY Wang S Estrov Z. Activation of a novel Bcr/Abl destruction pathway by WP1130 induces apoptosis of chronic myelogenous leukemia cells. Blood. (2007) 109:3470–8. 10.1182/blood-2006-02-005579 17202319 PMC1852235

[123] Li J Li H Zhu W Zhou B Ying J Wu J. Deubiquitinase inhibitor degrasyn suppresses metastasis by targeting USP5-WT1-E-cadherin signalling pathway in pancreatic ductal adenocarcinoma. J Cell Mol Med. (2020) 24:1370–82. 10.1111/jcmm.14813 31845546 PMC6991651

[124] Lee BH Lee MJ Park S Oh DC Elsasser S Chen PC. Enhancement of proteasome activity by a small-molecule inhibitor of USP14. Nature. (2010) 467:179–84. 10.1038/nature09299 20829789 PMC2939003

[125] Chen J Dexheimer TS Ai Y Liang Q Villamil MA Inglese J. Selective and cell-active inhibitors of the USP1/ UAF1 deubiquitinase complex reverse cisplatin resistance in non-small cell lung cancer cells. Chem Biol. (2011) 18:1390–400. 10.1016/j.chembiol.2011.08.014 22118673 PMC3344384

[126] Liu Y Lashuel HA Choi S Xing X Case A Ni J. Discovery of inhibitors that elucidate the role of UCH-L1 activity in the H1299 lung cancer cell line. Chem Biol. (2003) 10:837–46. 10.1016/j.chembiol.2003.08.010 14522054

[127] Wrigley JD Gavory G Simpson I Preston M Plant H Bradley J. Identification and characterization of dual inhibitors of the USP25/28 deubiquitinating enzyme subfamily. ACS Chem Biol. (2017) 12:3113–25. 10.1021/acschembio.7b00334 29131570

[128] Davis MI Pragani R Fox JT Shen M Parmar K Gaudiano EF. Small molecule inhibition of the ubiquitin-specific protease USP2 accelerates cyclin D1 degradation and leads to cell cycle arrest in colorectal cancer and mantle cell lymphoma models. J Biol Chem. (2016) 291:24628–40. 10.1074/jbc.M116.738567 27681596 PMC5114414

[129] Liang Q Dexheimer TS Zhang P Rosenthal AS Villamil MA You C. A selective USP1-UAF1 inhibitor links deubiquitination to DNA damage responses. Nat Chem Biol. (2014) 10:298–304. 10.1038/nchembio.1455 24531842 PMC4144829

[130] Okada K Ye YQ Taniguchi K Yoshida A Akiyama T Yoshioka Y. Vialinin A is a ubiquitin-specific peptidase inhibitor. Bioorg Med Chem Lett. (2013) 23:4328–31. 10.1016/j.bmcl.2013.05.093 23791076

[131] Lamberto I Liu X Seo HS Schauer NJ Iacob RE Hu W. Structure-guided development of a potent and selective non-covalent active-site inhibitor of USP7. Cell Chem Biol. (2017) 24:1490–500.e1411. 10.1016/j.chembiol.2017.09.003 29056421 PMC5749250

[132] Issaenko OA Amerik AY. Chalcone-based small-molecule inhibitors attenuate malignant phenotype via targeting deubiquitinating enzymes. Cell Cycle. (2012) 11:1804–17. 10.4161/cc.20174 22510564 PMC3372381

[133] Ratia K Pegan S Takayama J Sleeman K Coughlin M Baliji S. A noncovalent class of papain-like protease/deubiquitinase inhibitors blocks SARS virus replication. Proc Natl Acad Sci USA. (2008) 105:16119–24. 10.1073/pnas.0805240105 18852458 PMC2571001

